# Prebiotic supplementation modulates selective effects of stress on behavior and brain metabolome in aged mice

**DOI:** 10.1016/j.ynstr.2022.100501

**Published:** 2022-11-06

**Authors:** Joana S. Cruz-Pereira, Gerard M. Moloney, Thomaz F.S. Bastiaanssen, Serena Boscaini, Gabriel Tofani, Julia Borras-Bisa, Marcel van de Wouw, Patrick Fitzgerald, Timothy G. Dinan, Gerard Clarke, John F. Cryan

**Affiliations:** a Department of Anatomy and Neuroscience, University College Cork, Cork, Ireland; b APC Microbiome Ireland, University College Cork, Cork, Ireland; c Department of Psychiatry and Neurobehavioral Science, University College Cork, Cork, Ireland

**Keywords:** Aging, Prebiotics, Diet, Stress, Metabolome

## Abstract

Aging has a significant impact on physiology with implications for central nervous system function coincident with increased vulnerability to stress exposures. A number of stress-sensitive molecular mechanisms are hypothesized to underpin age-related changes in brain function. Recent cumulative evidence also suggests that aging impacts gut microbiota composition. However, the impact of such effects on the ability of mammals to respond to stress in aging is still relatively unexplored. Therefore, in this study we assessed the ability of a microbiota-targeted intervention (the prebiotic FOS-Inulin) to alleviate age-related responses to stress. Exposure of aged C57BL/6 mice to social defeat led to an altered social interaction phenotype in the social interaction test, which was reversed by FOS-Inulin supplementation. Interestingly, this occured independent of affecting social defeat-induced elevations in the stress hormone corticosterone. Additionally, the behavioral modifications following FOS-Inulin supplementation were also not coincident with improvement of pro-inflammatory markers. Metabolomics analysis was performed and intriguingly, age associated metabolites were shown to be reduced in the prefrontal cortex of stressed aged mice and this deficit was recovered by FOS-Inulin supplementation. Taken together these results suggest that prebiotic dietary intervention rescued the behavioral response to stress in aged mice, not through amelioration of the inflammatory response, but by restoring the levels of key metabolites in the prefrontal cortex of aged animals. Therefore, dietary interventions could be a compelling avenue to improve the molecular and behavioral manifestations of chronic stress exposures in aging via targeting the microbiota-gut brain axis.

## 1 Introduction

Aging is a complex multiorgan process that involves considerable molecular remodelling, which is characterized by defined hallmarks, such as mitochondrial dysfunction, loss of proteostasis and altered intercellular communication, which largely shapes the immune landscape due to time-dependent cellular damage accumulation (López-Otín et al., 2013). Inflammaging, the accumulation of pro-inflammatory signals that follow aging in mammals have been the subject of much investigation in the context of diverse age-related pathologies (Franceschi et al., 2018). Further, the allostatic load caused by the constant physiological adaptation in response to chronic stress (McEwen, 2007) is an important predictor of mortality (Robertson et al., 2017). Additionally, there are many neurobiological similarities between stress and aging that make it crucial to explore the effects of stress in the context of aging (Prenderville et al., 2015). These changes include hyperactivation of the hypothalamic–pituitary–adrenal (HPA) axis, deficiencies in neurotrophic factors such as brain-derived neurotrophic factor (BDNF), decreases in adult hippocampal neurogenesis, increased neuroinflammation, disruption of blood brain barrier, and changes in several neurotransmitters such as 5-hydroxytryptamine (5-HT or serotonin), noradrenaline, dopamine, glutamate, and γ-aminobutyric acid (GABA), all of which lead to neuronal dysfunction (Prenderville et al., 2015). Stress and HPA axis activation have been associated with a compromised profile of cognitive functions later in life (Qiu et al., 2021; Rimmele et al., 2022). Moreover, animal studies have highlighted a differential effect of chronic stress on dendritic spine remodelling in the prefrontal cortex (PFC) of aged compared to young rats (Bloss et al., 2011).

It is worth noting that the manifestation of anxiety and major depressive disorder is substantial in elderly adults, which significantly impacts their quality of life as a result of social isolation (Cacioppo and Hawkley, 2009; Prenderville et al., 2015). In humans, meta-analyses show that social networks tend to reduce with age and that novelty seeking, particularly relating to generating new social relationships, is reduced with age (Jopp et al., 2016; Prenderville et al., 2015). Even though social behavior has also been shown to be decreased in aged mice (Scott et al., 2017), this behavioral facet is still relatively unexplored in the context of aging (Prenderville et al., 2015). Taken together, the understanding of the impact of stress on/during aging is essential to provide solutions better suited for this particular demographic.

Throughout our lifespan, the gut microbiota – the millions of microbes that inhabit the gut – has been increasingly linked to the maintenance of homeostasis (Fung et al., 2017; Lynch and Pedersen, 2016; Miquel et al., 2018). Gut microbiome compositional changes have been reported with aging, namely a reduction of some commensal taxa (*ie Roseburia, Bifidobacterium* and *Prevotella)*, and an increase in other commensals (such as *Akkermansia* and *Christensenellaceae*) (Claesson et al., 2011; Ghosh et al., 2022). Furthermore, gut microbiomes from healthy individuals, while showing diverging time-dependent changes with age, are not only followed by increased circulation of specific microbial metabolites in the plasma, but also predict extended survival later in life (Wilmanski et al., 2021). These age-dependent gut microbiome alterations can reflect age-associated decline in health, but also suggest that lifestyle factors, particularly diet, offer an opportunity to shape the gut microbiome and hence contribute to better health outcomes (Ghosh et al., 2022). Diet has been shown to not only impact gut microbiome composition, but also predict and shape inflammation and mental health markers/status and cognitive function in aged human populations (Claesson et al., 2012; Ghosh et al., 2020).

The PFC is an important brain structure involved in emotional processing and social behavior (Franklin et al., 2017), also in response to social defeat (Challis et al., 2014; Covington et al., 2010), and is related to emotional regulation in aged humans (van Reekum et al., 2018; Winecoff et al., 2011). Further in aged rats, stress has been shown to impact neural oscillations in the PFC (Takillah et al., 2017). Interestingly, changes in the composition of the gut microbiome have been shown to drive microRNA expression in the PFC, along with transcriptional changes and regulation of the myelination process, thus implying a crucial role for the gut microbiome in the development and function of the PFC (Gacias et al., 2016; Alan E. Hoban et al., 2017; A. E. Hoban et al., 2016). Additionally, the gut microbiome has been implicated in the development, programming and expression of social behavior (Agranyoni et al., 2021; Desbonnet et al., 2014; Sherwin et al., 2019; Wu et al., 2021).

Diet plays a prominent role in shaping the gut microbiome, making it an accessible target for microbiota modulation (Audet, 2021; Sanders et al., 2019). Prebiotics, substrates that are used by gut microorganisms to confer health benefits to the host, are increasingly used as a dietary intervention to modulate the gut microbiome for health benefits (Sanders et al., 2019). Inulin, a polysaccharide dietary fiber, widely used as a prebiotic, has been shown to modulate the gut microbiome and thereby shape the immune system (Han et al., 2021; Zou et al., 2018). This prebiotic has been shown to modulate the gut microbiome in middle aged mice, reducing neuroinflammation and peripheral inflammation in response to stress (Boehme et al., 2020). We hypothesize that a prebiotic dietary intervention can modulate age-related changes in the gut microbiome to counter the effects of stress. We assess whether any potential effects are coincident with changes in the production of microbial and host metabolites in the prefrontal cortex and cecum.

## 2 Methods

### 2.1 Animals

Male aged C57BL/6 mice (n = 40; 18–19 months old; Charles River, Kent, UK) were used in this study. All experiments were conducted in accordance with European Directive 86/609/EEC, Recommendation 2007/526/65/EC, and approved by the Animal Experimentation Ethics Committee of University College Cork. Animals were kept under a 12-h light/dark cycle, with a temperature of 21 ± 1 °C and humidity of 55 ± 10%. Food and water were given *ad libitum*. Approximately one week before commencement of social defeat sessions, all mice were singly housed and weighed daily over the course of the experimental protocol. For the chronic social defeat stress procedure, non-experimental singly housed adult male CD1 mice (5 months old) were used as aggressors (Envigo, UK).

### 2.2 Study design

**Fig. 1:**
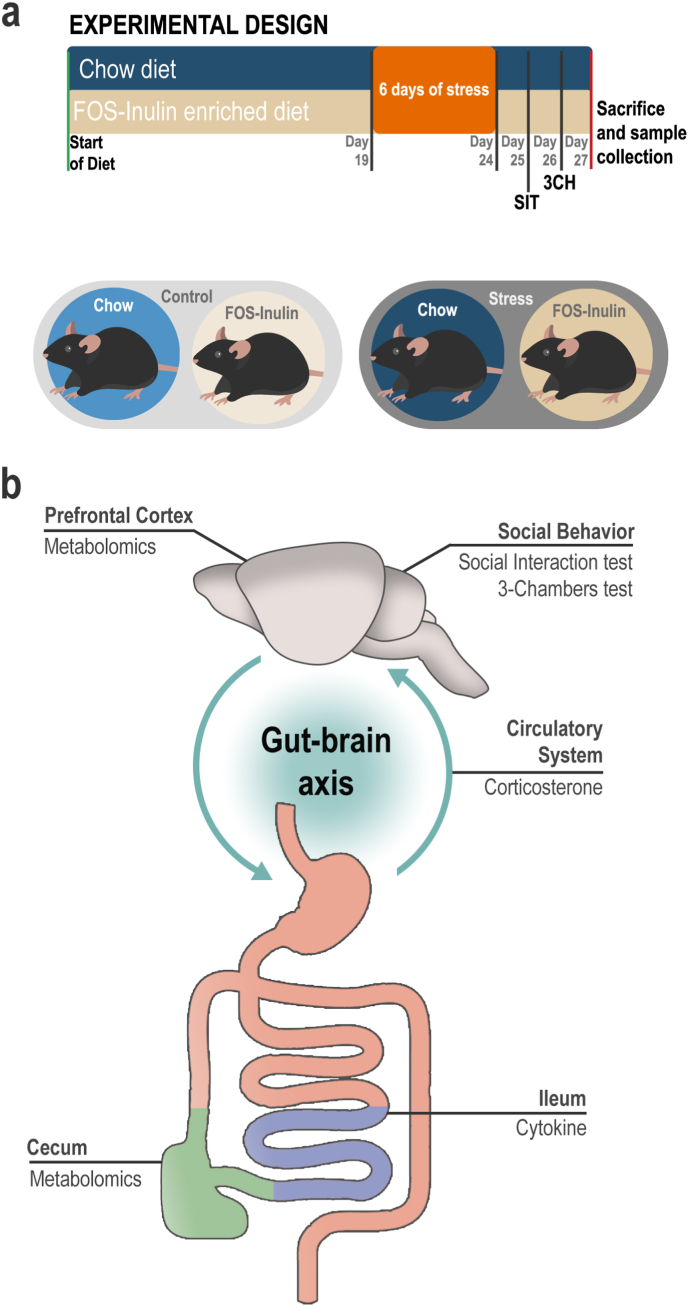
Experimental design. **a)** After approximately three weeks of FOS-Inulin dietary supplementation, animals were exposed to 6 days of social defeat stress. Subsequently, mice underwent social interaction test and 3-chambers sociability test, followed by sacrifice. **b)** Experimental outputs outlined in this experiment.

Aged C57BL/6 mice (19 months old) were fed either a FOS-Inulin enriched diet or chow diet for 19 days before the beginning of the stress protocol. For 6 days, all aged animals were exposed to social defeat stress (Fig. 1a). To understand if the prebiotic intervention acts on the gut brain axis to shape stress response in aged mice, we analysed social behavior using social interaction (Day 25) and the three-chamber test (Day 26). Tissues and biomarkers were harvested on day 27 including for measurement of circulating levels of corticosterone, ileal pro-inflammatory cytokine levels, and prefrontal cortex and cecal metabolomics (Fig. 1b).

### 2.3 Diet

Mice were fed chow (ssniff-Spezialdiäten GmbH, Soest, Germany) enriched with 10% FOS-Inulin (mixture of 92 ± 2% Inulin and 8 ± 2% Fructooligosaccharide, Orafti®Synergy 1; BENEO-Orafti N.V., Tienen, Belgium) or control chow (ssniff-Spezialdiäten GmbH, Soest, Germany) for 4 weeks, as previously described (Boehme, 2020).

### 2.4 Stress protocol

Mice were randomly assigned to either the stress (n = 20) or control groups (n = 20). Chronic social defeat stress was carried out daily for 6 consecutive days (see Fig. 1a for experimental timeline) as previously described but with slight modifications (Savignac et al., 2011). Prior to the defeat sessions, all CD1 aggressor mice were tested for aggressiveness over two separate days. A CD1 mouse was exposed to another CD1 mouse until the first attack. Mice with the shortest attack latencies were selected as aggressors to be used in subsequent social defeats. For each defeat session, experimental mice were exposed to a different aggressor CD1 mouse each day over the 6-day period. The session involved a single initial exposure of the test mouse to the aggressive CD1 in the home-cage of the aggressor (33 × 15 × 13cm) and lasted until the first attack with expression of submissive posturing, or until 5 min had passed. The latency to attack or display a submissive posture was recorded. The mice were then separated by a perforated Plexiglas® wall that allowed non-physical contact for 2 h. Then, the separator was removed and after another defeat, mice were returned to their home-cage. Control animals were left undisturbed in their homecages during the stress protocol.

### 2.5 Behavior

#### 2.5.1 Social interaction test

One day after the last social defeat session, the social interaction test was conducted as described before (A Gururajan et al., 2019). Briefly, it encompassed two trials of 150 s each. The test was performed in an open arena (40 × 32 × 24 cm, L × W × H) containing an empty wire mesh cage (9.5 × 7.5 × 7.0 cm) placed in the middle of one of the walls of the arena. During the first trial, the chamber in the social exploration box was empty; in the second trial an unfamiliar non-aggressive CD-1 male mouse was placed inside the exploration chamber in the arena. Both mice were returned to their home-cages upon completion of the test, and the arena was wiped with 70% ethanol. All mice were habituated 45 min before testing, and testing was conducted under red light (5 lux), being recorded from the ceiling. The interaction zone is defined as a rectangular area (25cmx15cm) around the wire-mesh cage where the CD1 target mouse is placed during the interaction phase. The corner zones are defined as squared areas on both corners opposite to the wire-mesh cage (9cmx9cm). Time spent in the interaction zone, time spent in the corner zones, movement and entries in the corner zones, and time spent facing the wire-mesh were scored using a deep-learning informed software analysis coupled with the SimBa 1.1.3, an open source toolkit for computer classification of complex social behaviors in experimental animals (Nilsson et al., 2020). Time facing the CD1 ratio was calculated as time spent facing the interaction zone during the second trial (CD1 target present) divided by the time spent facing the interaction zone during the first trial (CD1 target absent). DeepLabCut 2.2 with CUDA Toolkit 10.1 and Tensorflow 1.12.0 was used to perform behavioral analysis (Mathis et al., 2018). We defined 8-body part pose configuration and labelled 200–250 frames from representative videos from each group. The system was trained using a deep neural network that was trained in 155,000 iterations as the loss relatively flattened (Mathis et al., 2018; Nath et al., 2019). The trained network could accurately track the position of the mice in the full sets of video segments. The labelled x-axis (i.e. left–right) and y-axis (i.e. bottom-top) positions of the pixels in each frame were stored and exported in CSV format. Further analysis was performed using SimBa 1.1.3, where the width of the arena in centimeters was compared to the width in pixels, to generate a pixel-to-centimeter ratio (Nilsson et al., 2020). Next, we defined the region of interest analysis, as described above, and extracted the metrics calculated based on the X–Y coordinates of the center body part in a frame-by-frame basis. We followed the recommendations as described in https://github.com/sgoldenlab/simba.

#### 2.5.2 3-Chamber test

Social cognition was evaluated using the 3-chamber social interaction test, in which time spent interacting with a novel conspecific is compared to time spent with a novel object or familiar conspecific. It is based on the premise that mice will prefer to seek an animal over an inanimate object, and that they will prefer a novel conspecific to a familiar one. This test was performed 24 h after the social interaction test. Mice were habituated to the room for 1 h before testing.

The test arena consisted of 3 chambers; the left and right chambers measured 13.5 × 20 × 20 cm and the center chamber was 9 × 20 × 20 cm. A solid partition divided the chambers, with a small hole allowing access to the other chambers. There were 3 trials in this test: habituation, sociability, and social novelty preference. All phases of the test lasted 10 min, were performed sequentially, and recorded from above for later analysis. During the habituation phase, the mouse was placed into the center chamber and then allowed access to the empty left and right chambers for 10 min. The mouse was then gently coaxed to the center chamber and a novel mouse was placed in a mesh cage in one of the side chambers, whereas a novel object (a small rubber duck) was placed in a mesh cage in the other side chamber for the sociability trial. Placement of the novel mouse and novel objects were randomized between animals to eliminate side preferences. For the social novelty trial, an aged-matched novel mouse was placed in the mesh cage that had previously housed the novel object. The 3-chamber apparatus was cleaned with 70% ethanol between animal trials. The animals were habituated to the room for 45 min before the test, and the test was conducted under dim light (60 lux). The time spent in each chamber was then scored using DeepLabCut and SimBa, as described in the previous section.

### 2.6 Tissue collection

One day after the conclusion of behavioral testing, the animals were sacrificed. Animals were killed by decapitation in a random fashion regarding testing groups between 09.00 h and 15.00 h. Trunk blood was collected in EDTA-containing tubes and centrifuged for 15 min at 10,000 g at 4 °C. Plasma was collected and stored at −80 °C for later analysis. Whole cecum and ileum were removed and snap-frozen on dry ice and stored at −80 °C. Brain tissue was rapidly hand-dissected and snap frozen in dry ice and then stored at −80 °C until further tissue processing. Spleens and mesenteric lymph nodes (MLNs) were dissected out of the animals and were processed for flow cytometry as described in the corresponding section.

### 2.7 Prefrontal cortex and cecal metabolomics

The cecal and prefrontal cortex metabolome was analysed by MS-Omics as follows. Prefrontal cortex and cecal content were acidified using hydrochloric acid, and deuterium labelled internal standards were added. All samples were analysed in a randomized order. Analysis was performed using a high polarity column (Zebron™ ZB-FFAP, GC Cap. Column 30 m × 0.25 mm x 0.25 μm) installed in a GC (7890 B, Agilent) coupled with a quadropole detector (5977 B, Agilent). The system was controlled by ChemStation (Agilent). Raw data was converted to netCDF format using Chemstation (Agilent), before the data was imported and processed in Matlab R2014b (Mathworks, Inc.) using the PARADISe software described by Johnsen et. al. (Johnsen et al., 2017).

Peaks were quantified using area under the curve (AUC). Biostatistics were run in R (version 4.1.2) with the Rstudio GUI (version 1.4.1717). Principal-component analysis was performed on CLR-transformed values (Aitchison et al., 2000). The PERMANOVA implementation from the vegan library was used to find structural differences between treatments on a compositional level. To find metabolites that were differentially abundant based on either stress or prebiotic supplementation, we fitted linear models using the CLR-transformed metabolite levels with both factors as explanatory variables. Linear models were also used to test for concordance or discordance of metabolite levels between the prefrontal cortex and cecum, again including stress and prebiotic as additional explanatory variables. In order to assess differences between singular pairs of groups we used Tukey's HSD procedure. To correct for multiple testing (FDR) in tests involving metabolomics features, Storey's q-value posthoc procedure was performed with a q-value of 0.2 as a cut-off (Storey, 2002). Custom scripts to analyze data can be found online at https://github.com/thomazbastiaanssen/Tjazi (Bastiaanssen et al., 2022). Metabolomics figures were generated using ggplot2.

### 2.8 Cytokine quantification

The levels of secreted interleukin-1β (IL-1β), interferon gamma (IFN-γ), IL-5, IL-6, IL-10 (not detected), tumor necrosis factor-α (TNFα) and CXCL1 (chemokine (C-X-C motif) ligand 1) were analysed with the Pro-inflammatory Panel 1 (mouse) V-PLEX Kit and the MESO QuickPlex SQ 120, SECTOR Imager 2400 (Meso Scale Discovery, Maryland, USA). Only data derived from duplicates with <15% CV were included in the analysis. Concentrations of cytokines were expressed in pg/mg of tissue. Limits of detection are represented in Supplementary Table 1.

### 2.9 Plasma corticosterone quantification

Corticosterone quantification of plasma (15 μL) collected from trunk blood at the sacrifice was performed using a corticosterone ELISA (Enzo Life Sciences) and was performed according to the manufacturer's instructions and was analysed as previously described (Bastiaanssen et al., 2021; Anand Gururajan et al., 2022). A multi-mode plate reader (Synergy HT, BioTek Instruments) was used to quantify light absorbance in the assay, at 405 nm. Only data derived from duplicates with <15% CV were included in the analysis. Concentrations of plasma corticosterone were expressed in ng/mL. Limit of detection is represented in Supplementary Table 1.

### 2.10 Cell isolation and flow cytometry

Spleens and mesenteric lymph nodes (MLNs) were dissected out of the animal, cleaned from fat tissue and stored in media (RPMI-1640 medium with L-glutamine and sodium bicarbonate - R8758, Sigma), supplemented with 10% FBS (F7524l, Sigma) and 1% Pen/strep (P4333, Sigma) on wet ice for flow cytometry the same day.

Flow cytometry was performed as previously described (Boehme et al., 2020; A Gururajan et al., 2019). Spleenocytes were isolated by flushing the spleen with media using a syringe. The cell suspension was subsequently centrifuged, aspirated and incubated with 1 mL lysis buffer (Sigma, R7757) for 5 min 10 mL media was added to dilute the lysis buffer and the cell suspension was poured over a 70 μm strainer, after which it was centrifuged and aspirated. 2 × 10^6^ cells were resuspended in 90 μL staining buffer and split into 2 aliquots for the staining procedure. MLNs were transferred onto a 70 μm strainer and disassembled using the plunger of a 1 mL syringe. The strainer was subsequently rinsed with 10 mL media, and the cell suspension was centrifuged, aspirated, 2 × 10^6^ cells were resuspended in 90 μL staining buffer, and split into 2 aliquots for the staining procedure.

For the staining procedure, 5 μL of FcR blocking reagent (Miltenyi, 130-092-575) was added to each sample. Samples were subsequently incubated with a mix of antibodies (Supplementary Table 2) for 30 min on ice, after which they were centrifuged, aspirated and fixed using 100 μL 4% PFA for 30 min on ice. Samples were finally centrifuged, aspirated and resuspended in staining buffer for flow cytometric analysis the following day on the BD FACSCalibur. Data was analysed using FlowJo (Version 10).

### 2.11 Statistical analysis

All data are represented as mean ± SEM. Statistical analyses were conducted using SPSS 27 (IBM, USA). Normality was assessed employing the Shapiro–Wilk test and for equality of variances using the Levene's test. Non-parametric data were analysed with independent-samples Kruskal–Wallis test followed by pairwise comparisons adjusted by the Bonferroni correction for multiple tests, with a 95% confidence interval. Parametric data were analysed using two-way analysis of variance (ANOVA) and pairwise comparisons were assessed using a Tukey HSD adjustment. Time spent in interaction area, time spent in the corners, movement in the corners, entries in corners and social novelty were analysed using a general linear model integrating stress, diet and stimulus. Further, we utilized simple main effects to explore the individual group differences in these complex models, adjusted by the Bonferroni correction for multiple tests, as we hypothesized a priori that the stress-only group would be significantly different from the other 3 groups. Statistical significance was set at p ≤ 0.05.

## 3 Results

### 3.1 Prebiotic supplementation reverses behavioral impairments but not endocrine effects of social defeat stress in aged animals

**Fig. 2:**
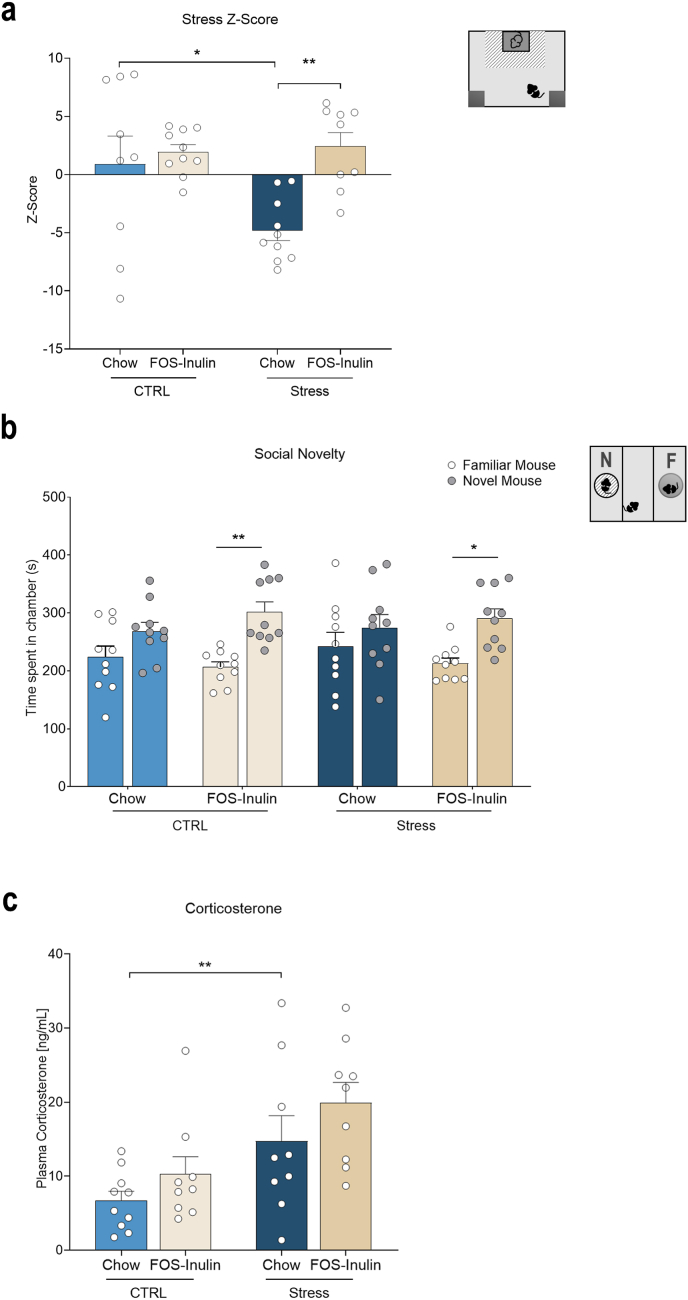
**– FOS-Inulin supplementation ameliorates the effects of stress in aged mice. a)** Integrative Z-score of social interaction outputs; Stressed animals fed with Chow have a significantly reduced Z-score compared to control animals, which is restored by FOS-Inulin supplementation. **b)** FOS-Inulin supplemented mice, but not chow-fed animals, spend significantly more time in the novel mouse chamber. **c)** Plasma corticosterone is higher in aged animals fed with chow when exposed to stress, which is not recovered in stressed animals supplemented with FOS-Inulin. Results presented as mean + standard error of the mean (SEM). n = 9–10 per group. *p < 0.05, **p < 0.01.

**Supplementary Figure 1:**
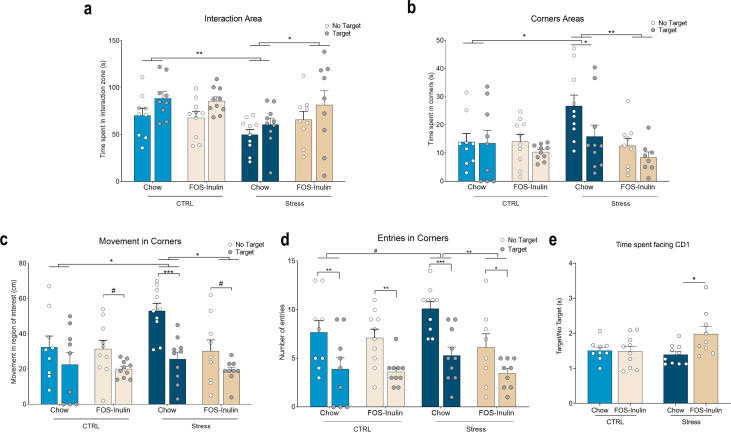
FOS-Inulin supplementation effects on stress response in the social interaction test in aged mice. a) Time spent in the interaction area. Stress-Chow animals spend significantly less time than their controls in the interaction zone. **b)** Time spent in the corner zones. Stressed animals fed with FOS-Inulin spend significantly less time in the corners than stressed animals fed with chow. **c)** Movement in corners – FOS-Inulin supplementation significantly reduces the movement in corners in aged animals exposed to stress (Stress-Chow vs Stress-Inulin). **d)** Entries in corners – all animals show less entries in the corner zones when the CD-1 is present in the arena. **e)** Time spent by the experimental animal facing the CD1 is significantly increased in stressed animals supplemented with FOS-Inulin. Results presented as mean + standard error of the mean (SEM). n = 8–10 per group. **p < 0.01, ***p < 0.001

Following psychosocial stress, animals were tested in the social interaction test to assess the behavioral response of aged mice to stress. Stress significantly reduced the time spent in the interaction zone in Chow-fed animals (F_(1,34)_ = 5.820, p = 0.021, CTRL-Chow vs Stress-Chow p = 0.005, Supplementary Fig. 1a), which was rescued by FOS-Inulin supplementation (Stress-Chow vs Stress–FOS–Inulin; p = 0.027). An effect of diet was observed in time spent in the corner zones (F_(1,33)_ = 6.650, p = 0.015), and simple main effects showed stressed animals supplemented with FOS-Inulin spent significantly less time in the corners than stressed animals fed with chow (Stress-Chow vs Stress–FOS–Inulin; p = 0.003, Supplementary Fig. 1b). Movement inside the corner zones showed a significant effect of diet (F_(1,34)_ = 4.594, p = 0.039), as demonstrated by Stress–FOS–Inulin animals moving significantly less in the corners than Stress-Chow animals (p = 0.011, Supplementary Fig. 1c). Further, with regards to entries into the corner zone, there was a significant effect of diet (F_(1,33)_ = 5.217, p = 0.029) as FOS-Inulin supplementation in stressed animals significantly reduced entries in the corners (Stress-Chow vs Stress–FOS–Inulin, p = 0.009, Supplementary Fig. 1d). Considering a ratio of the time spent by the experimental animal facing the CD-1/interaction zone, there was an overall interaction effect of stress and diet (F_(1,37)_ = 4.703, p = 0.037) as revealed by an increase in the time facing the CD-1 by stressed animals supplemented with FOS-Inulin when compared to stressed controls (Stress-Chow vs Stress–FOS–Inulin, p = 0.028, Supplementary Fig. 1e). To further understand how our interventions shaped stress-responding in the mice, we created a z-score combining the time spent in the interaction zone when the CD1 is present, time spent in the corner zones in presence and absence of CD1, the ratio of time spent facing the interaction zone, the entries and movement in the corner zones, and the movement in the interaction area. Two-way ANOVA revealed an interaction effect of stress and diet (F_(3,37)_ = 4.958, p = 0.033), as revealed by a significant decrease in the Z-score in the Stress-Chow group compared to the non-stressed Control-Chow group (p = 0.031) and followed by an amelioration of the stress phenotype by FOS-inulin supplementation, as shown by an increase in the FOS-Inulin group in comparison to the Stress-Chow group (p = 0.004, Fig. 2a).

To assess the influence of FOS-Inulin supplementation on social behavior in stressed aged animals, mice were tested in the three-chamber apparatus. While an overall significant interaction effect of stress and diet (F_(1,36)_ = 1.364, p = 0.250) was not observed, there was a significant overall preference between a novel and familiar mouse (F_(1,36)_ = 14.914, p < 0.001), which was not observed in animals fed with Chow (Control-Chow: p = 0.175; Control–FOS–Inulin: p = 0.006; Stress-Chow: p = 0.328, Stress–FOS–Inulin: p = 0.021, Fig. 2b).

To assess the endocrine response to stress, plasma levels of corticosterone were assessed. Corticosterone in plasma collected at the time of sacrifice showed a significant group effect (H (3) = 15.226, p = 0.002). Corticosterone was elevated in Stress-Chow compared to Control-Chow (H = −17.167, p. adj = 0.003), which is not reversed by FOS-Inulin supplementation (H = 3.667, p. adj = 1, Fig. 2c).

### 3.2 FOS-INULIN increases ileal pro-inflammatory cytokines

**Fig. 3:**
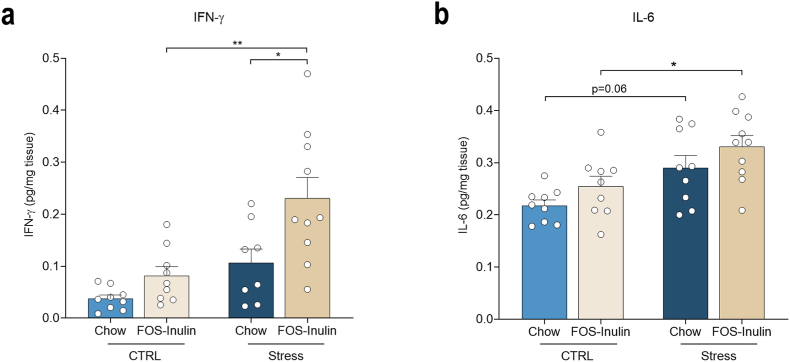
**– FOS-Inulin supplementation increases pro-inflammatory cytokines in the ileum. a)** FOS-Inulin supplementation significantly increases the concentration of IFN-γ in the ileum of stressed aged animals, when compared with stressed animals fed with chow and control animals fed with FOS-Inulin; **b)** Stressed aged animals show increased IL-6 concentration in the ileum (non-significantly), which is further inflated by FOS-Inulin supplementation. Results presented as mean + standard error of the mean (SEM). n = 8–10 per group. *p < 0.05, **p < 0.01, ***p < 0.001.

**Supplementary Figure 2:**
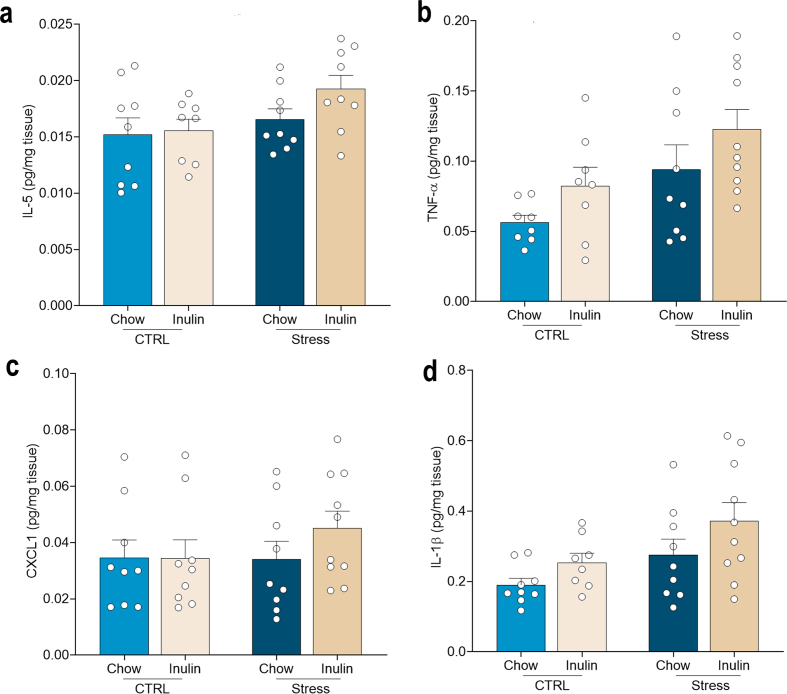
Stress does not impact further ileal cytokine levels. a) FOS-Inulin supplementation shows a trend for increased IL-5 in the ileum of stressed aged animals, when compared with control animals fed with chow. **b)** TNF-α is significantly increased in stressed animals with FOS-Inulin supplementation, when compared with control animals fed with chow. Stress and diet did not impact the levels of CXCL1 **(c)** or IL-1β **(d)** in the ileum of aged mice. Results presented as mean + standard error of the mean (SEM). n = 7–10 per group. **p < 0.01

To understand how FOS-inulin supplementation impacts immune outputs in stressed aged mice, cytokine levels were assessed by MSD in the ileum, as this particular small intestine region is important in the context of immune and cytokine response to dietary factors (Toubal et al., 2020). IFN-γ is a pro-inflammatory cytokine, previously linked to social behavior (Filiano et al., 2016), which is increased in aged mice (Baruch et al., 2014) in the CNS, which was found to be increased by stress exposure (F_(1,35)_ = 16.154, p = 0.000332) and diet (F_(1,35)_ = 9.653, p = 0.004), though Two-Way ANOVA showed no interaction effects (F_(1,35)_ = 2.175, p = 0.150). IFN-γ was significantly increased in stressed animals supplemented with FOS-Inulin, when compared to mice treated with FOS-Inulin alone (Stress–FOS–Inulin vs Control–FOS–Inulin, p = 0.002) and when compared to control stressed animals (Stress–FOS–Inulin vs Stress-Chow, p = 0.014, Fig. 3a), thus suggesting that stress and diet are driving IFN-γ concentrations independently. IL-6 is a pro-inflammatory cytokine that increases with age, while associated with poor cognitive function and predicts mortality in the elderly (Puzianowska-Kuźnicka et al., 2016). Ileal concentrations of IL-6 were impacted by stress (F_(1,36)_ = 14.563, p = 0.001), as stressed animals fed with Chow show an increased tendency in comparison to control counterparts (Stress-Chow vs Control-Chow, p = 0.064), however this was unaltered by FOS-Inulin supplementation (Stress–FOS–Inulin vs Stress-Chow, p = 0.454), as FOS-Inulin supplementation increased IL-6 levels in stressed animals, when compared to their controls (Stress–FOS–Inulin vs Control–FOS–Inulin, p = 0.04, Fig. 3b). Notably, FOS-Inulin supplementation seems to exacerbate the levels of IFN-γ and IL-6 in the ileum of aged stressed animals. Furthermore, while IL-5 shows an effect of stress (F_(1,31)_ = 4.644, p = 0.039), there is only a non-significant trend for increased IL-5 in stressed animals supplemented with FOS-Inulin (Stress–FOS–Inulin vs Control-Chow, p = 0.083, Supplementary Fig. 2a). Moreover, TNF-α concentrations show a significant effect of group (H (3) = 11.110, p = 0.011), as FOS-Inulin supplementation increases TNF-α ileal levels when compared to the control group (Stress–FOS–Inulin vs Control-Chow, H = −16.0175 p. adj = 0.006, Supplementary Fig. 2b). Finally, CXCL1 and IL-1β did not show a group effect (CXCL1: H (3) = 3.082, p = 0.379; IL-1β: H (3) = 7.733, p = 0.052, Supplementary Figs. 2c and 2d).

**Supplementary Figure 3:**
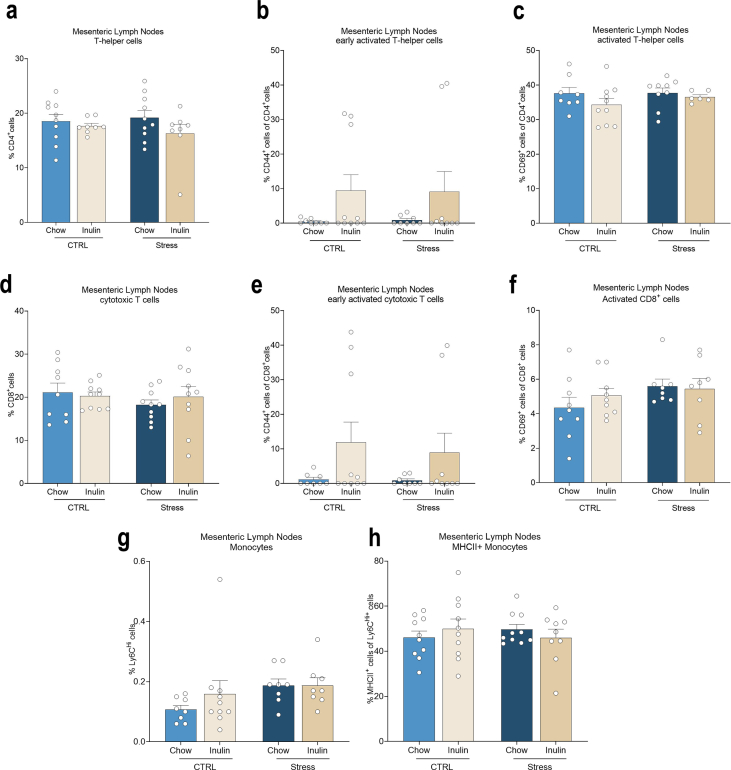
Flow cytometry analysis of general populations in the mesenteric lymph nodes reveals no impact caused by stress or diet. a) Percentage of CD4^+^ cells, b) percentage of CD4^+^CD44^+^ cells, c) percentage of CD4^+^CD69^+^ cells, d) percentage of CD8^+^ cells, e) percentage of CD8^+^CD44^+^ cells, f) percentage of CD8^+^CD69^+^ cells, g) percentage of Ly6CHi + cells, h) percentage of Ly6CHi + MHCII + cells. Results presented as mean + standard error of the mean (SEM). n = 8–10 per group.

**Supplementary Figure 4:**
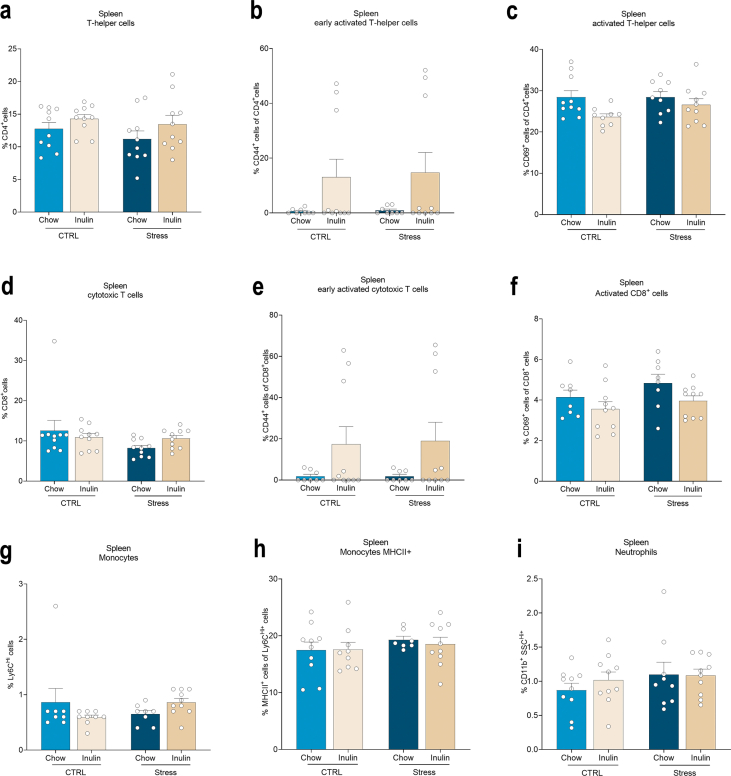
Flow cytometry analysis of general populations in the spleen reveals no impact caused by stress or diet. a) Percentage of CD4^+^ cells, b) percentage of CD4^+^CD44^+^ cells, c) percentage of CD4^+^CD69^+^ cells, d) percentage of CD8^+^ cells, e) percentage of CD8^+^CD44^+^ cells, f) percentage of CD8^+^CD69^+^ cells, g) percentage of Ly6CHi + cells, h) percentage of Ly6CHi + MHCII + cells, i) percentage of CD11b + SSCHi cells. Results presented as mean + standard error of the mean (SEM). n = 8–10 per group.

Flow cytometry in mesenteric lymph nodes and spleen of general immune populations did not reveal any involvement of the peripheral immune system in the FOS-Inulin supplementation in response to stress in aging (Supplementary Figs. 3 and 4).

### 3.3 Inulin rescues metabolite levels in the prefrontal cortex of aged stressed animals

**Fig. 4:**
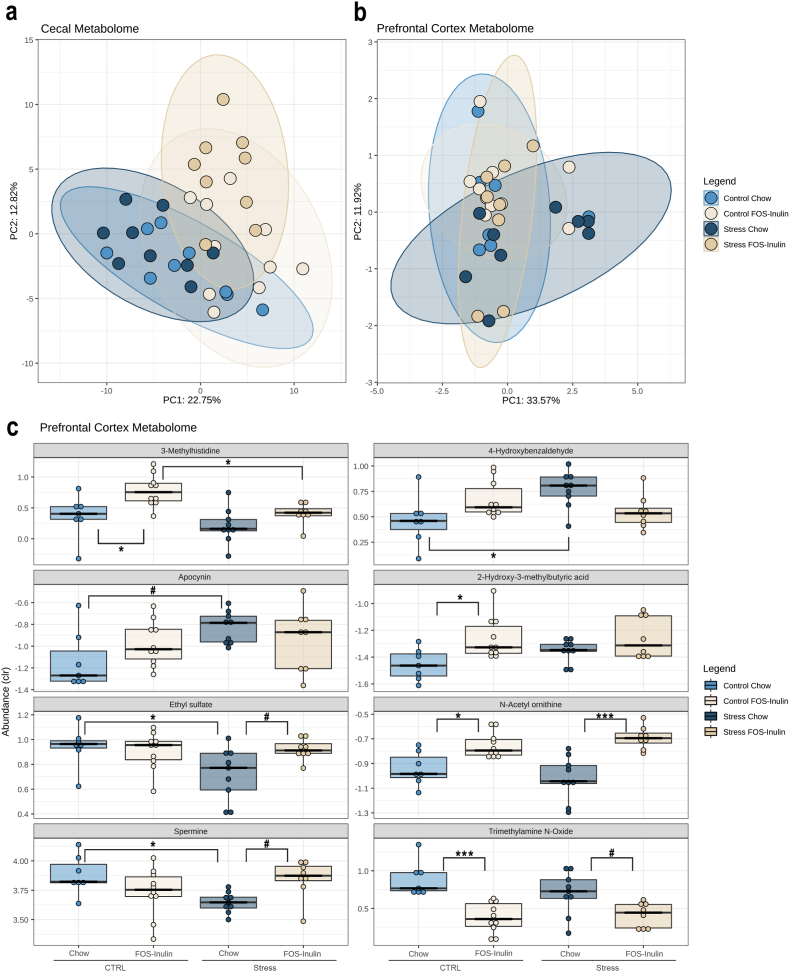
**– Spermine and 4-Hydroxybenzaldehyde are modulated by FOS-Inulin supplementation in the prefrontal cortex of aged-stressed animals.** a-b) Principal component analysis showing the main effect of diet in the metabolome of the cecum a) and main effect of stress in the b) prefrontal cortex of stressed aged mice. c) Boxplots showing the centered log-ratio transformed (clr) abundance of prefrontal cortex metabolites that displayed a significant effect of stress, diet, or an interaction between the two. In the prefrontal cortex, 8 metabolites were altered by stress and/or diet – metabolites were altered in the PFC of stressed aged mice, which were restored in FOS-Inulin supplemented animals. For the boxplots, boxes represent the limits of the interquartile range, the horizontal line represents the median point, and the whiskers represent the full data range. n = 7–10 per group. #p < 0.1, *p < 0.05, **p < 0.01, ***p < 0.001.

To understand if gut microbial-derived metabolites could possibly drive the positive prebiotic response to stress in aged mice, metabolomics analysis was performed in the cecum and prefrontal cortex of stressed and non-stressed aged mice. In total, 205 metabolites were identified in the cecum and 105 were detected in the PFC. In the cecum, diet significantly impacted the cecum metabolome, whereas in the PFC, stress was the main clustering factor (Fig. 4a and b). In the cecum, 139 metabolites were found altered by diet in the cecum, but no stress or interaction effects were found (Supplementary Table 3). In the prefrontal cortex, 8 metabolites were altered by stress or diet after post-hoc correction: 3-Methylhistidine, 4-Hydroxybenzaldehyde, apocynin, 2-hydroxy-3-methylbutyric acid, ethyl sulfate, N-acetyl ornithine, spermine and trimethylamine N-oxide. Of these, only 4-Hydroxybenzaldehyde and spermine show an interaction effect of stress and diet (Fig. 4c). 4-Hydroxybenzaldehyde is a naturally occurring benzaldehyde that has been shown to boost antioxidant activity and wound healing (Chen et al., 2021; Kang et al., 2017) – despite showing an interaction effect of stress and diet (β = −0.42, p = 0.003, q = 0.0994), as this metabolite is increased in the PFC of Stress-Chow animals (Stress-Chow vs Control-Chow, p = 0.019), FOS-Inulin supplementation does not significantly reverse this increase in stressed aged animals (p = 0.109). Spermine, a natural polyamine reported to modulate autophagy-related age effects (Xu et al., 2020), also presented an interaction effect of stress and diet (β = 0.349, p = 0.004, q = 0.0994) as it was found to be decreased in the prefrontal cortex of stressed aged animals (Stress-Chow vs Control-Chow p = 0.036) and presents a non-significant tendency to be recovered by FOS-Inulin supplementation (Stress–FOS–Inulin vs Stress-Chow, p = 0.072).

Further, stress impacted the prefrontal cortex concentration of apocynin (β = 0.308, p = 0.011, q = 0.178), as stressed aged mice show a tendency for increase of apocynin (Stress-Chow vs Control-Chow p = 0.054), which is not reduced by FOS-Inulin supplementation (Stress–FOS–Inulin vs Stress-Chow, p = 0.746). Likewise, ethyl sulfate concentrations were also affected by stress (β = −0.223, p = 0.009, q = 0.178), as reflected by a reduction in ethyl sulfate in the PFC of stressed aged mice (Stress-Chow vs Control-Chow p = 0.045), which shows a tendency to be partially restored by FOS-Inulin supplementation (Stress–FOS–Inulin vs Stress-Chow, p = 0.066).

Additionally, FOS-Inulin supplementation significantly impacted the concentrations of 3-Methylhistidine (β = 0.411, p = 0.004, q = 0.178), as it was increased in control animals on a FOS-Inulin supplemented diet (Control–FOS–Inulin vs Control-Chow, p = 0.02) and interestingly reduced by stress exposure (Stress–FOS–Inulin vs Control–FOS–Inulin, p = 0.03). Likewise, 2-hydroxy-3-methylbutyric acid was significantly altered by diet (β = 0.197, p = 0.006, q = 0.980), as it was increased in the PFC of mice on a FOS-Inulin supplemented diet in control animals (Control–FOS–Inulin vs Control-Chow, p = 0.03) and in animals exposed to stress (Stress–FOS–Inulin vs Control-Chow, p = 0.03). Moreover, N-acetyl ornithine was also significantly affected by diet (β = 0.189, p = 0.006, q = 0.165), since FOS-Inulin supplementation increased concentrations of this metabolite in PFC of control (Control–FOS–Inulin vs Control-Chow, p = 0.03) and stressed animals (Stress–FOS–Inulin vs Stress-Chow, p = 7.72 × 10^−5^). Similarly, trimethylamine N-oxide concentrations in the PFC were largely impacted by diet (β = −0.5136, p = 6.73 × 10^−5^, q = 0.007), since FOS-Inulin supplementation reduces trimethylamine N-oxide in control animals (Control–FOS–Inulin vs Control-Chow, p = 0.0004) and presents a trend for reduction in stressed animals (Stress–FOS–Inulin vs Stress-Chow, p = 0.06).

In summary, stress mainly altered the concentration of apocynin and ethyl sulfate in the prefrontal cortex, while FOS-Inulin supplementation mainly changed the concentration of 3-Methylhistidine, 2-hydroxy-3-methylbutyric acid, and trimethylamine N-oxide, while 4-Hydroxybenzaldehyde and spermine show an interaction effect of stress and diet. This suggests that this dietary intervention modulates the levels of spermine and 4-Hydroxybenzaldehyde, in the prefrontal cortex of stressed aged mice, posing as a potential new avenue to explore age-dependent stress effects.

## 4 Discussion

Some underexplored aspects of aging are the mechanisms underlying the response to stress, and its consequent behavioral outcomes. In this study, we demonstrate a clear interaction between a dietary intervention targeting the microbiome in modulating the stress response in aged mice. In particular, the present study demonstrates that FOS-Inulin supplementation improves social interaction in response to stress in aged mice and regulates spermine and 4-Hydroxybenzaldehyde concentrations in the prefrontal cortex.

In line with previous reports from our group (Scott et al., 2017), aged mice exhibit social novelty deficits in contrast to their young counterparts. In this study, we further observed that stress did not exacerbate social novelty deficits, but that FOS-Inulin supplementation improved overall social recognition in aged mice, thus implying that a prebiotic dietary intervention in aging can mitigate age-dependent behavioral deficits. Social defeat exposure also elevated plasma corticosterone levels – however, prebiotic supplementation did not result in the reversal of the corticosterone levels, suggesting a dissociation between the impact of the prebiotic intervention on the behavioral and physiological response to stress.

Given the cumulative evidence that the gut microbiome is intertwined with age-related peripheral and neuroinflammation (Boehme et al., 2020; Claesson et al., 2012; Scott et al., 2017), we explored the potential mediation of age-related effects by inflammatory factors to understand if the FOS-Inulin supplementation effects were related to inflammatory factors. In this study, analysis of pro-inflammatory cytokines was focused on the ileum, as this region of the small intestine is important for the sampling of antigens from diet, hence influencing the immune system (Brown and Esterházy, 2021). Contrary to our expectations, FOS-Inulin supplementation was found to increase the concentration of pro-inflammatory cytokines in the small intestine of stressed animals. A few reports have described that FOS and inulin supplementation can increase inflammation in the colon of mice in an immune dysregulated colitis model (Singh et al., 2019). Given that aging induces a low-grade inflammatory profile, and that aging interferes with the resolution of acute inflammation (Arnardottir et al., 2014), it is not completely surprising that the prebiotic intervention could provoke moderate inflammation in the small intestine of these animals. Further, the exacerbation of pro-inflammatory markers by FOS-Inulin supplementation on stressed animals could be a reflection of a ‘double hit’ effect as stress is a known inducing factor of inflammatory markers (Cruz-Pereira et al., 2020; Marsland et al., 2017). Surprisingly, in peripheral immune structures, namely the mesenteric lymph nodes and spleen, we found no changes to general immune cell populations, suggesting that the behavioral outcomes are not related to extra-intestinal modulation of inflammation by stress or FOS-Inulin (Supplementary Figs. 2 and 3).

It has previously been reported that FOS-Inulin dietary intervention shapes gut microbiome in middle-aged mice (Boehme et al., 2020), and that prebiotic fibers are known to modulate the gut microbiome (O'Connor et al., 2020), and further that the metabolome is intrinsically connected with the gut microbiome (Garza et al., 2020; Valles-Colomer et al., 2019). Metabolomics were performed in the cecum and prefrontal cortex to profile the microbial metabolite levels in an intestinal structure that is reshaped by dietary fibers (Drew et al., 2018) and an important brain region for emotional processing and social behavior altered in aging (Franklin et al., 2017; Yan and Rein, 2022). As expected, diet was the main factor to shape the cecal metabolome, which follows consistent observations that dietary fibers change the gut microbiota in humans (Le Bastard et al., 2020; Vandeputte et al., 2017) and in rodents (Drew et al., 2018; Fuhren et al., 2021). Curiously, in an aged human cohort, despite the reshaping of the gut microbiota, inulin intake did not impact immune cell numbers in the peripheral blood (Kiewiet et al., 2021), which aligns with our results (Supplementary Figs. 2 and 3).

In the PFC, 4 metabolites were altered by the prebiotic supplementation alone, that may play an important role in the context of aging: 3-methyl-L-histidine, acetylornithine, trimethylamine-N-oxide and 2-hydroxy-3-methylbutyric acid. In dementia patients, 3-methyl-L-histidine has been found declined in the blood, and is associated with frailty (Teruya et al., 2021), while its supplementation suppresses glial inflammation and ameliorates neurovascular-unit dysfunction in an aged Alzheimer's disease model (Kaneko et al., 2017), and in this study is increased with the prebiotic supplementation. Curiously, acetylornithine, also positively modulated by prebiotic supplementation in our study, has also been found elevated in the serum of dementia patients (Weng et al., 2019), while being reduced in the cerebral cortex of aged mice (Ding, 2021). Trimethylamine-N-oxide, which in this study was reduced by prebiotic supplementation, is increased in both elderly humans and aged mice, and can accelerate brain cellular senescence, while enhancing cognitive impairment (Li et al., 2018). Lastly, 2-Hydroxy-3-Methylbutyric acid was identified as the metabolite responsible for the promotion of intestinal epithelial cell proliferation upon *Lactobacillus paracasei* probiotic administration (Qiao et al., 2022), and is increased by FOS-Inulin supplementation in the prefrontal cortex. While it is remarkable that the levels of these metabolites in the prefrontal cortex are affected by prebiotic supplementation and have previously been reported to be involved in aging, the functional consequences of these alterations remain unclear as well as the underlying mechanisms.

In our analysis, we found that stress alone independently affected only two metabolites in the prefrontal cortex of aged animals after *post-hoc* correction: apocynin and ethyl sulfate. Apocynin suppresses reactive oxygen species, partially reversing the aging process in mesenchymal stem cells and boosts osteogenesis (Sun et al., 2015), and in this study is found increased in the prefrontal cortex of aged animals exposed to stress. Ethyl sulfate is a metabolite classically linked to ethanol degradation (Helander and Beck, 2005), but to the best of our knowledge this is still underexplored in the context of brain metabolome.

Here, we further show that the combination of prebiotic dietary intervention and social defeat stress in aged mice, is associated with alterations in the levels/abundance of two metabolites in the prefrontal cortex: spermine and 4-Hydroxybenzaldehyde. Spermidine, the precursor of spermine, has been found to extend the lifespan by promoting autophagy (Aman et al., 2021). Spermine and spermidine delay aging-related cognitive impairments by enhancing autophagy and mitochondrial function in the brain of a mouse model of accelerated aging (Xu et al., 2020). Further, spermine and spermidine levels have been found to be enhanced in the blood of centenarians (Pucciarelli et al., 2012). Here, we found that levels of spermine in the aged prefrontal cortex are reduced by stress exposure, and that the prebiotic intervention recovered the spermine levels. Spermine has been shown to prevent LPS-induced memory deficits (Frühauf-Perez et al., 2018), and has been found to be slightly reduced in the brains of rats after restraint stress (Hayashi et al., 2004). Furthermore, spermine is also an NMDA receptor agonist, and has been shown to be increased in the PFC of aged rats (Gupta et al., 2012). Given that aging has been linked to reduced NMDA receptor function (Gramuntell et al., 2021), it is feasible to associate elevated CNS spermine levels with age-dependent cognitive decline. Taken together, this could potentially suggest that stress in aged mice may increase NMDA receptor function potentially through the reduction of spermine concentration, which in turn can result in excitotoxicity, in a similar fashion to what is observed in Alzheimer's disease (Wang and Reddy, 2017). Further studies are needed to dissect this hypothesis.

4-Hydroxybenzaldehyde is a relatively underexplored compound isolated from *Gastrodia elata*, a common Chinese herbal medicine that has been shown to boost acute wound healing and is altered in the plasma of middle-aged mice of a short-longevity model (Kadota et al., 2021). In this study, FOS-Inulin reduced the levels of this metabolite upon stress exposure. It is challenging to draw objective conclusions from this observation, as the functional properties of this particular metabolite still remains mostly unexplored – however, it is possible that it could represent an interesting target for future experiments (or dietary modulation) as there are still a considerable number of metabolites that remain unidentified (Bar et al., 2020). Taken together with the relative novelty of 4-Hydroxybenzaldehyde in this context, further studies dissecting the involvement of these metabolites in age-dependent effects will be crucial.

## 5 Conclusions

In conclusion, this study provides evidence that prebiotic supplementation with FOS-Inulin ameliorates the stress response in aged mice at a behavioral and metabolic level. This was not associated with modulation of the immune system or HPA axis but through the regulation key age-associated metabolites, spermine and 4-Hydroxybenzaldehyde, in the PFC. Further studies targeting spermine and 4-Hydroxybenzaldehyde and their metabolic pathways will be essential to uncover the specific effects and mechanisms underpinning the impact of these metabolites on the age-related stress-induced behavioral phenotypes.

## Ethics approval and consent to participate

Animal experiments were conducted with the approval and oversight of the Animal Experimentation Ethics Committee of University College Cork.

## Availability of data and materials

The datasets used and/or analysed during the current study are available from the corresponding author on reasonable request.

## Funding

10.13039/100016205APC Microbiome Ireland is a research center funded by 10.13039/501100001602Science Foundation Ireland (SFI/12/RC/2273_P2). Prof. Cryan is funded by 10.13039/501100001602the Science Foundation Ireland (SFI/12/RC/2273_P2), Saks Kavanaugh Foundation and 10.13039/100000001Swiss National Science Foundation project CRSII5_186,346/NMS 2068, and has received research funding from 4D Pharma, Cremo, Dupont, 10.13039/100006628Mead Johnson, Nutricia, and Pharmavite; has been an invited speaker at meetings organized by 10.13039/501100011031Alimentary Health, Alkermes, Ordesa, and Yakult; and has served as a consultant for Alkermes and Nestle. Prof. Clarke has received honoraria from Janssen, Probi, and Apsen as an invited speaker; is in receipt of research funding from 10.13039/100007082Pharmavite and Fonterra; and is a paid consultant for Yakult, Zentiva and Heel pharmaceuticals. Ms. Cruz-Pereira was also funded by the 10.13039/501100001592HEA Covid-19 Cost Extension Fund.

## CRediT authorship contribution statement

**Joana S. Cruz-Pereira:** Conceptualization, Formal analysis, Investigation, Writing – original draft. **Gerard M. Moloney:** Investigation, Writing – review & editing. **Thomaz F.S. Bastiaanssen:** Formal analysis, Data curation. **Serena Boscaini:** Investigation. **Gabriel Tofani:** Investigation, Visualization. **Julia Borras-Bisa:** Investigation. **Marcel van de Wouw:** Investigation. **Patrick Fitzgerald:** Resources. **Timothy G. Dinan:** Supervision. **Gerard Clarke:** Supervision, Writing – review & editing. **John F. Cryan:** Supervision, Writing – review & editing.

## Declaration of competing interest

The authors declare that they have no known competing financial interests or personal relationships that could have appeared to influence the work reported in this paper.

## Data Availability

Data will be made available on request.
